# Supplementary material for: Synthesis and Characterization of Radiogallium-Labeled Cationic Amphiphilic Peptides as Tumor Imaging Agents
Source: Cancers (Basel). 2021 May 14;13(10):2388. doi: 10.3390/cancers13102388 (PMC8155856; doi:10.3390/cancers13102388)
Supplement: Supplementary file 1 [file cancers-13-02388-s001.zip › cancers-1162866-supplementary.pdf]

Supplementary Material: Synthesis and Characterization of Radiogallium-Labeled Cationic Amphiphilic Peptides as Tumor Imaging Agents

Takeshi Fuchigami, Takeshi Chiga, Sakura Yoshida, Makoto Oba, Yu Fukushima, Hiromi Inoue, Akari Matsuura, Akira Toriba and Morio Nakayama

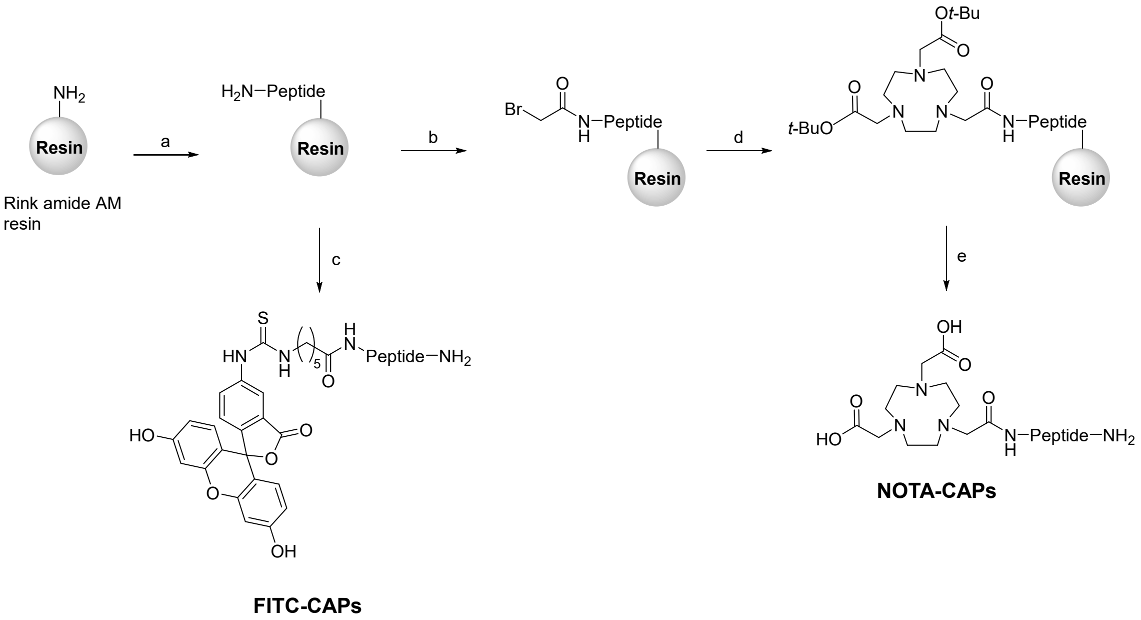

Scheme S1. Synthetic scheme of FITC-CAPs and NOTA-CAPs. Reagents and conditions: (a) (1) Fmoc-amino acid, 1-[bis(dimethylamino)methylene]-1H-benzotriazolium 3-oxide hexafluorophosphate (HBTU), 1-hydroxybenzotriazole (HOBt), N,N-diisopropylethylamine (DIPEA), *N,N*-dimethylformamide (DMF), RT, 30 min, (2) 20% piperidine in DMF RT, 15 min, (3) cycles of (1) and (2); (b) bromoacetic acid, 1-ethyl-3-(3-dimethylaminopropyl)carbodiimide, CH_2Cl_2, RT, 1 h; (c) (1) Fmoc-6-aminohexanoic acid, HBTU, HOBt, DIPEA, DMF, RT, 30 min, (2) 20% piperidine in DMF, RT, 15 min, (3) fluorescein isothiocyanate, DIPEA, DMF, 4) trifluoroacetic acid (TFA)/H2O/triisopropyl silane (TIS)/1,2-ethanedithiol (EDT), RT, 90 min; (d) 1) 1,4,7-triazacyclononane, CH_2Cl_2, RT, 3 h, 2) *tert*-butyl-2-bromoacetate, DIPEA, 1-methyl-2-pyrrolidone, RT, 2 h; (e) TFA/H_2O/TIS/EDT, RT, 90 min.

Table S1. Sequence and MALDI-TOF MS data of CAPs.

Peptides	Calculated MS $(M+H)^+$	Observed MS $(M+H)^+$
FITC-KV4	2080.27	2081.32
FITC-KV6	2535.3	2535.8
FITC-KV8	2989.63	2598.65 (–FITC)
FITC-RV6	2759.4	2758.3
FITC-HV6	2607.0	2606.0
NOTA-KV6	2318.0	2317.9
NOTA-RV6	2542.2	2541.3
NOTA-HV6	2389.8	2388.4
Ga-NOTA-RV6	2610.0	2610.8
Ga-NOTA-HV6	2457.5	2454.8
Ga-NOTA-KV6	2407.7	2407.9

Table S2. In vitro stability of ^{67}Ga-NOTA-CAPs in PBS and mouse plasma.

^{67}Ga-NOTA-CAPs	In Vitro Stability	
	PBS (24 h)	Mouse Plasma (1 h)
^{67}Ga-NOTA-KV6	98.1 (1.18)	75.7 (5.58)
^{67}Ga-NOTA-RV6	99.0 (0.43)	27.4 (2.94)

^{67}Ga-NOTA-HV6	97.1 (0.78)	23.3 (0.75)

The in vitro stability of ^{67}Ga-NOTA-CAPs in phosphate buffered saline (PBS) and mouse plasma was evaluated using HPLC. Data are expressed as the percentage of remaining intact radiotracers. Each value represents the mean (SEM) of three individual experiments.

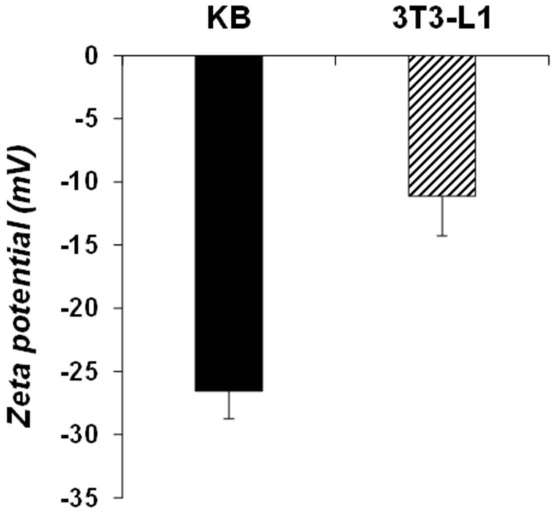

Figure S1. Zeta potential of KB cells and 3T3-L1 cells (5.0 × 10^5 cells/mL) in phosphate buffered saline. Values are mean ± SEM (n = 3).

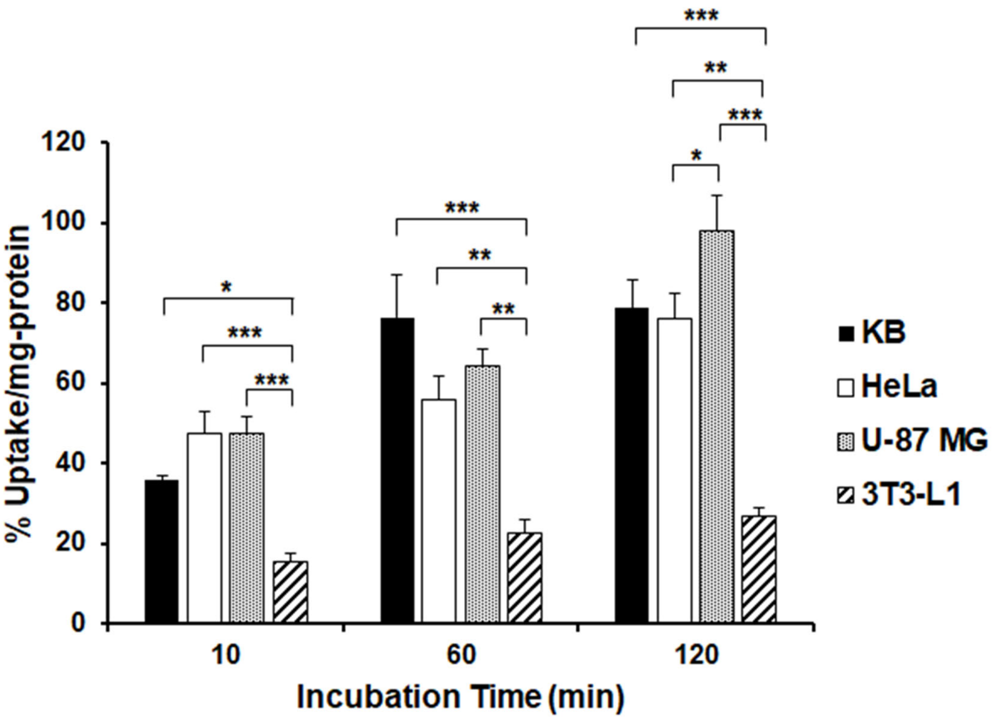

Figure S2. Cellular uptake of ^{67}Ga-NOTA-KV6 in cancer cells (KB, HeLa, and U-87 MG) and fibroblast 3T3-L1 cells. * $p < 0.05$, ** $p < 0.01$, *** $p < 0.001$ compared with 3T3-L1 cells. (ANOVA Tukey's multiple comparison test). Values are mean ± SEM (n = 4-7).

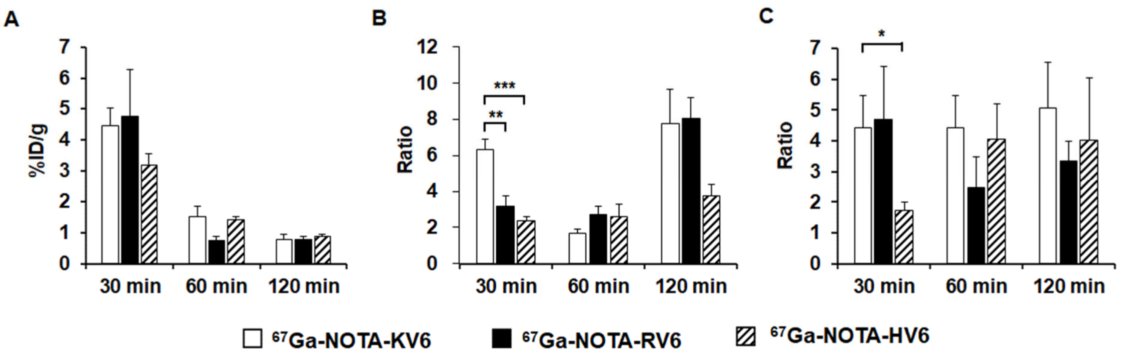

Figure S3. Comparison of in vivo tumor uptake (**A**), tumor-to-blood ratios (**B**), and tumor-to-muscle ratios (**C**) of ^{67}Ga-NOTA-KV6, ^{67}Ga-NOTA-RV6, and ^{67}Ga-NOTA-HV6 in KB tumor-bearing mice at each time point. Values are mean ± SD (n = 4-6). * $p < 0.05$, ** $p < 0.01$, *** $p < 0.001$ (ANOVA, Tukey t test).
